# Machine Learning Models for Predicting Cycloplegic Refractive Error and Myopia Status Based on Non-Cycloplegic Data in Chinese Students

**DOI:** 10.1167/tvst.13.8.16

**Published:** 2024-08-09

**Authors:** Bole Ying, Rajat S. Chandra, Jianyong Wang, Hongguang Cui, Julius T. Oatts

**Affiliations:** 1 Lower Merion High School, Ardmore, PA, USA; 2 University of Pennsylvania Perelman School of Medicine, Philadelphia, PA, USA; 3 Department of Ophthalmology, The First Affiliated Hospital, College of Medicine, Zhejiang University, Hangzhou, P. R. China; 4 Department of Ophthalmology, University of California San Francisco, San Francisco, CA, USA

**Keywords:** machine learning, myopia, refractive error, prediction, cycloplegic refraction

## Abstract

**Purpose:**

To develop and validate machine learning (ML) models for predicting cycloplegic refractive error and myopia status using noncycloplegic refractive error and biometric data.

**Methods:**

Cross-sectional study of children aged five to 18 years who underwent biometry and autorefraction before and after cycloplegia. Myopia was defined as cycloplegic spherical equivalent refraction (SER) ≤−0.5 Diopter (D). Models were evaluated for predicting SER using *R*^2^ and mean absolute error (MAE) and myopia status using area under the receiver operating characteristic (ROC) curve (AUC). Best-performing models were further evaluated using sensitivity/specificity and comparison of observed versus predicted myopia prevalence rate overall and in each age group. Independent data sets were used for training (n = 1938) and validation (n = 1476).

**Results:**

In the validation dataset, ML models predicted cycloplegic SER with high *R*^2^ (0.913–0.935) and low MAE (0.393–0.480 D). The AUC for predicting myopia was high (0.984–0.987). The best-performing model for SER (XGBoost) had high sensitivity and specificity (91.1% and 97.2%). Random forest (RF), the best-performing model for myopia, had high sensitivity and specificity (92.2% and 96.9%). Within each age group, difference between predicted and actual myopia prevalence was within 4%.

**Conclusions:**

Using noncycloplegic refractive error and ocular biometric data, ML models performed well for predicting cycloplegic SER and myopia status. When measuring cycloplegic SER is not feasible, ML may provide a useful tool for estimating cycloplegic SER and myopia prevalence rate in epidemiological studies.

**Translational Relevance:**

Using ML to predict cycloplegic refraction based on noncycloplegic data is a powerful tool for large, population-based studies of refractive error.

## Introduction

The prevalence of myopia is growing worldwide, and is already at epidemic levels in some countries, particularly among the younger generations of East and Southeast Asia.^1^ The global number of individuals with myopia is predicted to reach 4.8 billion by 2050.^1^ In children, the prevalence of myopia increases with age until age 18, with young adult myopia rates as high as 70% to 90% in some parts of China.^2^ Myopia can be associated with several vision-threatening conditions including myopic maculopathy, optic neuropathy, and retinal detachment.^3^ Thus closely monitoring myopia prevalence, severity, and progression to high myopia is warranted at both individual and population levels to reduce the burden of myopia.

Refractive error measured after cycloplegia is the gold standard for detecting myopia and quantifying myopia severity.^4^ However, measuring cycloplegic refractive error can be challenging in children, particularly in large population-based epidemiology studies, which require enormous time and resources. Non-cycloplegic refractive error is still commonly used for determining the presence or severity of myopia,^5^^–^^8^ despite its well-documented tendency to overestimate myopia prevalence and severity. Previous studies consistently demonstrated substantial differences between cycloplegic and noncycloplegic refractive error measurements, with mean spherical equivalent differences ranging from 0.60 to 1.23 D,^9^^–^^14^ suggesting noncycloplegic refractive error overestimating the prevalence and severity of myopia in the pediatric population. Thus strategies to improve myopia detection and the classification of myopia severity under noncycloplegic conditions are needed.

Attempts have been made to develop prediction models for cycloplegic refractive error using measurements obtained without cycloplegia.^15^^–^^22^ These prediction models are based on traditional linear regression or logistic regression models that use various predictors including demographics, ocular biometric measures, noncycloplegic refractive error and uncorrected visual acuity (UCVA). These prediction models have been variably successful (*R*^2^ ranging from 0.26 to 0.93), likely because of variations in the selected predictors, children's ages, and their refractive error.^15^^–^^21^

In recent years, machine learning (ML) models, including random forest, lasso, and gradient boosting models, have demonstrated great potential for application in ophthalmology and vision research,^23^^–^^25^ including our previous works for predicting treatment burden and vision response to anti-vascular endothelial growth factor (anti-VEGF) treatment for neovascular age-related macular degeneration.^26^^,^^27^ In contrast to traditional regression models that usually assume the linear relationship between predictors and outcome, ML models can accommodate complex and nonlinear relationships between predictors and outcome and thus have great potential to improve prediction performance.

In this study, we aim to develop and validate ML models for predicting cycloplegic refractive error and myopia status using easily obtainable measures under noncycloplegic conditions in large number of Chinese school students aged five to 18 years from two cities in China. The models included demographics, noncycloplegic refractive error, UCVA, and ocular biometric measurements. We hypothesized that ML models can accurately predict cycloplegic refractive error and myopia status, thus providing a useful tool to determine cycloplegic refractive error and myopia status for a child, and myopia prevalence rate in populations where cycloplegic refractive error cannot be obtained.

## Methods

This is a secondary analysis of data from a cross-sectional school-based study of myopia conducted in two cities (Jinyun, Hangzhou) of Zhejiang province, China. Details of the study have been reported in previous publications.^12^^,^^28^^,^^29^ In brief, from October 2020 to January 2021, students aged five to 18 years were enrolled from schools in Jinyun (rural; n = 1938) and Hangzhou (urban; n = 1498). Human subject research approval was obtained from Zhejiang University and the local Administration of the Education and School Board. Written informed consent was obtained from each participant's legal parent or guardian. The study followed the tenets of the Declaration of Helsinki.

### Eye Examinations and Ocular Biometry

All study participants underwent a comprehensive eye examination by an optometrist or ophthalmologist. This included UCVA testing with a retro-illuminated logMAR chart with tumbling-E optotypes and ocular biometry using the NIDEK A-scan (Nidek, Tokyo, Japan) under noncycloplegic conditions (biometric parameters recorded included AL, CR, central corneal thickness, and anterior chamber depth). Participants also underwent autorefraction before and after cycloplegia using a table-mounted NIDEK autorefractor (Model ARK-510A; Nidek). For cycloplegic autorefraction, one drop of 0.5% tropicamide was instilled in each eye every five minutes for four administrations and cycloplegic refractive error was taken 30 minutes after the last administration. For both noncycloplegic and cycloplegic refractive error, three readings were taken from each eye. If the difference between any of two readings of sphere or cylinder from an eye was greater than 0.5 D, the refractive error measurement was repeated. For each eye, the average of three readings of refractive error was used for statistical analysis.

### Machine Learning Models

We applied ML models for predicting two outcome measures of cycloplegic refractive error in each eye including (1) cycloplegic spherical equivalent refraction (SER) calculated as sphere + 0.5 * cylinder; (2) myopia defined as cycloplegic SER ≤−0.5 D considering the negative sign (i.e., −0.5 D or worse). The predictors for the ML models were: demographics (age, gender), wearing refractive correction (yes/no), eye laterality (left eye, right eye), noncycloplegic SER, biometric measures (AL, CR, AL/CR ratio, central corneal thickness, and anterior chamber depth), intraocular pressure and UCVA (in logMAR). To improve the generalizability of ML model performances, two large independent datasets were used. The dataset from 1938 students in Jinyun was used for training models through cross-validation, and the dataset from 1476 students in Hangzhou was used for independently validating models (i.e., for testing models).

We evaluated six ML models for predicting cycloplegic SER: (1) support-vector machine (SVM), (2) random forest, (3) extreme gradient boosting (XGBoost), (4) multilayer perceptron (MLP) neural network, (5) linear regression, and (6) lasso regression. For predicting myopia status, we evaluated the same ML models excluding linear regression and lasso regression because these models are not as applicable for direct classification analysis. We implemented these ML models because they are among the most commonly used and, with the exception of the linear and lasso models, are all appropriate for making predictions for both regression (e.g., for continuous outcome) and classification (e.g., for discrete outcome) tasks. We have previously applied these models for predicting the number of pro re nata anti-VEGF injections and vision responses to anti-VEGF treatment for neovascular age-related macular degeneration.^26^^,^^27^

The SVM model determines a separation between different classes and predicts a continuous outcome with high generalization ability by transforming data to high-dimensional spaces.^30^ Random forest models make predictions by combining the results of a collection of tree predictors while avoiding overfitting even with the addition of more trees.^31^ The XGBoost model is similar to the random forest model in that it uses an ensemble of tree predictors, but uses gradient tree boosting and is trained in an additive manner.^32^ The MLP model is a supervised neural network that starts with an input layer consisting of the given predictors as a set of nodes, ultimately producing an output for classification or regression after multiple transformations of values from previous layers with a weighted linear summation and nonlinear activation function.^33^ We used the linear regression and lasso regression models only for the regression analysis, as they are relatively simple, with lasso regression serving as an interpretable model because it can reduce the number of non-zero coefficients.^34^

We tuned the hyperparameters for each of the models through 10-fold cross-validation using the training dataset, which helps avoid overfitting as the ML models learn how to predict the desired outcomes. We tuned hyperparameters of the regression models for predicting cycloplegic SER by optimizing *R*^2^ (a measure for quantifying the amount of variation in the outcome explained by the predictors). For the classification models for predicting myopia status, we tuned the hyperparameters by optimizing the F1 score (the harmonic mean of recall and precision). After tuning the hyperparameters through 10-fold cross-validation, the ML model was fit on the entire training dataset using these hyperparameters. Once trained, the ML models were evaluated on the validation dataset. The primary measures for evaluating model performance in the training and validation datasets were *R*^2^ and mean absolute error (MAE) for predicting cycloplegic SER of an eye, and accuracy and the area under the receiver operating characteristic (ROC) curve (AUC) for predicting myopia status of an eye. To quantify the importance of each feature used in the predictions, we used the permutation importance, defined by the decrease in model's *R*^2^ for predicting cycloplegic SER and the decrease in the model's AUC for predicting myopia status after shuffling each feature.^31^ Feature importance was assessed in both the training and validation datasets.

We used Python 3.9 and its open-source package scikit-learn version 1.1.3 to implement the ML models.^35^ The code for our ML analysis can be provided upon reasonable request to the authors. We selected the best-performing ML models (based on the model performance in the validation dataset) for further statistical analysis. Using the prediction output from the best-performing ML model for predicting cycloplegic SER and the best-performing ML model for predicting myopia status, we calculated (1) the difference between predicted and observed cycloplegic SER overall, by age group, and by level of cycloplegic SER; (2) the predicted and observed myopia prevalence rate overall and by age group; and (3) sensitivity and specificity and their 95% confidence intervals (95% CI) for predicting myopia status. In these analyses, predicted person-level myopia positive was defined as predicted cycloplegic SER −0.5 D or worse in either eye from the ML model for predicting cycloplegic SER, or myopia positive in either eye from the ML model for predicting myopia status. These statistical analyses were performed in SAS v9.4 (SAS Institute Inc, Cary, NC, USA).

## Results

The training dataset included 3876 eyes from 1938 students in Jinyun. The validation dataset included 2951 eyes from 1476 students in Hangzhou, after excluding 22 students with missing data. Demographic and ocular characteristics of students in the training and validation datasets are summarized in Table 1. The age of the students ranged from five to 18 years, with a mean (SD) of 9.6 (3.6) years in the training dataset and 9.9 (3.6) years in the validation dataset. Females accounted for 51.5% in the training dataset and 49.6% in the validation dataset. Mean cycloplegic SER was −0.07 (2.11) D in the training dataset and −0.35 (2.26) D in the validation dataset. The percent of eyes with myopia was 29.6% in the training dataset and 36.6% in the validation dataset. The mean noncycloplegic SER was −1.1 (1.92) D in the training dataset and −1.2 (2.04) D in the validation dataset, with 51.1% and 53.7% of eyes having noncycloplegic SER ≤−0.5 D, respectively. UCVA of 20/200 or worse was found in 5.3% of eyes in the training dataset and 8.1% of eyes in the validation dataset, whereas 59.7% eyes in the training dataset and 36.4% eyes in the validation dataset had UCVA of 20/20 or better. Ocular biometric measurements were very similar between the training and validation datasets (respectively): AL 23.5 (1.3) mm and 23.6 (1.3) mm, CR 7.83 (0.25) mm and 7.84 (0.26) mm, AL/CR ratio 3.00 (0.14) and 3.01 (0.16), central corneal thickness 555 (31) µm and 549 (31) µm, anterior chamber depth 3.61 (0.32) mm and 3.56 (0.30) mm, intraocular pressure 17.2 (2.8) mm Hg and 17.6 (2.9) mm Hg.

**Table 1.** Characteristics of Study Participants in the Training and Validation Datasets

Demographic Characteristics	Training Dataset From Jinyun (N = 1938 Participants)	Validation Dataset From Hangzhou (N = 1476 Participants)
Age (yrs)		
5	35 (1.8%)	23 (1.6%)
6	429 (22.1%)	225 (15.2%)
7	317 (16.4%)	263 (17.8%)
8	210 (10.8%)	216 (14.6%)
9	148 (7.6%)	145 (9.8%)
10	132 (6.8%)	69 (4.7%)
11	148 (7.6%)	91 (6.2%)
12	109 (5.6%)	81 (5.5%)
13	82 (4.2%)	83 (5.6%)
14	69 (3.6%)	57 (3.9%)
15	65 (3.4%)	54 (3.7%)
16	72 (3.7%)	57 (3.9%)
17	61 (3.1%)	58 (3.9%)
18	61 (3.1%)	54 (3.7%)
Mean (SD)	9.6 (3.6)	9.9 (3.6)
Female sex	999 (51.5%)	732 (49.6%)
Wearing refractive correction	439 (22.7%)	302 (20.5%)
Ocular characteristics	N = 3876 eyes	N = 2951 eyes
Cycloplegic SER (diopters)		
≤−6.0	67 (1.7%)	72 (2.4%)
>−6.0, ≤−3.0	371 (9.6%)	321 (10.9%)
>−3.0, ≤−0.5	711 (18.3%)	686 (23.2%)
>−0.5, ≤0.5	739 (19.1%)	556 (18.8%)
>0.5, ≤3.0	1930 (49.8%)	1271 (43.1%)
>3.0	58 (1.5%)	45 (1.5%)
Mean (SD)	−0.07 (2.11)	−0.35 (2.26)
Non-cycloplegic SER (diopters)		
≤−6.0	97 (2.5%)	92 (3.1%)
>−6.0, ≤−3.0	499 (12.9%)	411 (13.9%)
>−3.0, ≤−0.5	1386 (35.8%)	1081 (36.6%)
>−0.5, ≤0.5	1480 (38.2%)	1011 (34.3%)
>0.5, ≤3.0	390 (10.1%)	345 (11.7%)
>3.0	24 (0.6%)	11 (0.4%)
Mean (SD)	−1.1 (1.92)	−1.2 (2.04)
Uncorrected visual acuity		
20/200 or worse	206 (5.3%)	240 (8.1%)
>20/200–20/100	272 (7.0%)	263 (8.9%)
>20/100–20/50	356 (9.2%)	364 (12.3%)
20/40	148 (3.8%)	156 (5.3%)
20/33	214 (5.5%)	258 (8.7%)
20/25	367 (9.5%)	597 (20.2%)
20/20 or better	2313 (59.7%)	1073 (36.4%)
Axial length (mm), mean (SD)	23.5 (1.3)	23.6 (1.3)
Corneal curvature radius (mm), mean (SD)	7.83 (0.25)	7.84 (0.26)
Axial length/corneal curvature radius ratio, mean (SD)	3.00 (0.14)	3.01 (0.16)
Anterior chamber depth (mm), mean (SD)	3.61 (0.32)	3.56 (0.30)
Central corneal thickness (µm), mean (SD)	555 (31)	549 (31)
Intraocular pressure (mm Hg), mean (SD)	17.2 (2.8)	17.6 (2.9)

### Machine Learning Models for Predicting Cycloplegic SER

We evaluated six ML models for predicting cycloplegic SER in each eye. In the training dataset, all six ML models predicted cycloplegic SER well (Table 2, online Supplementary Figure S1.1) with *R*^2^ ranging from 0.922 (lasso) to 0.994 (random forest), and MAE of 0.121 D (random forest) to 0.431 D (lasso). In the training dataset, the random forest model performed best (*R*^2^ = 0.994, MAE = 0.121 D) and XGBoost performed second best (*R*^2^ = 0.974, MAE = 0.256 D). In the validation dataset (Table 2, online Supplementary Figure S1.2), the XGBoost model performed best (*R*^2^ = 0.935, MAE = 0.393 D), SVM was second best (*R*^2^ = 0.934, MAE = 0.402 D), and the lasso model performed worst (*R*^2^ = 0.913, MAE = 0.480 D). The importance of features in each ML model in the training dataset and validation dataset is shown in online Supplementary Figures S2.1 and S2.2. Noncycloplegic SER, AL, AL/CR ratio and UCVA were consistently among the top four most important features for predicting cycloplegic SER in all six models except linear regression model where AL was replaced by CR as one of the top four most important features.

**Table 2.** Performance of Machine Learning Models for Predicting Cycloplegic Refractive Error in the Training and Validation Datasets

Model	Training (N = 1938 Students, 3876 Eyes)	Validation (N = 1476 Students, 2951 Eyes)
*R*^2^, mean (SD)		
SVM	0.951 (0.003)	0.934 (0.005)
Random forest	0.994 (0.000)	0.933 (0.004)
XGBoost	0.974 (0.001)	0.935 (0.005)
MLP	0.927 (0.003)	0.917 (0.005)
Linear	0.927 (0.003)	0.917 (0.005)
Lasso	0.922 (0.003)	0.913 (0.004)
Absolute error, mean (SD)		
SVM	0.332 (0.005)	0.402 (0.008)
Random forest	0.121 (0.002)	0.413 (0.008)
MLP	0.423 (0.006)	0.468 (0.008)
Linear	0.423 (0.006)	0.469 (0.008)
Lasso	0.431 (0.006)	0.480 (0.009)

Standard deviation (SD) is determined from 1000-fold bootstrapping.

### Machine Learning Models for Predicting Myopia Status

We evaluated four ML models for predicting myopia status in each eye. Their performance results in the training and validation datasets are reported in Table 3. In the training dataset, the 4 ML models performed excellently for predicting myopia status with accuracy ranging from 0.965 (MLP) to 1.000 (XGBoost) and AUC ranging from 0.994 (SVM and MLP) to 1.000 (XGBoost) (Table 3, online Supplementary Figure S3.1). The XGBoost model performed best (accuracy and AUC both equal to 1), and the random forest model performed second best (accuracy of 0.980 and AUC of 0.998). In the validation dataset, the random forest model performed best with accuracy of 0.953 and AUC of 0.987, and the XGBoost model performed second best with an accuracy of 0.952 and with AUC of 0.984 (Table 3, online Supplementary Figure S3.2). The importance of features identified in each of these 4 ML models is shown in online Supplementary Figures S4.1 and S4.2. Noncycloplegic SER, AL, AL/CR ratio and UCVA were again consistently among the top four features for predicting myopia status.

**Table 3.** Performance of Machine Learning Models for Predicting Myopia Status in the Training and Validation Datasets

Model	Training (N = 1938 Students, 3876 Eyes)	Validation (N = 1476 Students, 2951 Eyes)
Accuracy mean (SD)		
SVM	0.966 (0.003)	0.935 (0.005)
Random forest	0.980 (0.002)	0.953 (0.004)
XGBoost	1.000 (0.000)	0.952 (0.004)
MLP	0.965 (0.003)	0.947 (0.004)
AUC mean (SD)		
SVM	0.994 (0.001)	0.984 (0.002)
Random forest	0.998 (0.000)	0.987 (0.002)
XGBoost	1.000 (0.000)	0.984 (0.002)
MLP	0.994 (0.001)	0.984 (0.002)

SD is determined from 1000-fold bootstrapping.

### The Best-Performing Model for Predicting Cycloplegic SER by Age Group and Refractive Error Group

We further assessed model performance for XGBoost, the best-performing ML model for predicting cycloplegic SER, by evaluating its performance for subsets based on age and magnitude of cycloplegic SER. In the training dataset, the overall mean difference (SD) between predicted and observed cycloplegic SER was 0.00 (0.34) D (95% limits of agreement of −0.66 D to 0.66 D, Table 4). For all age group strata, the mean difference between predicted and observed cycloplegic SER was within 0.07 D and the mean absolute difference within 0.29 D for each age group. When evaluating differences between predicted and observed based on magnitude of cycloplegic SER, the model did best with moderate myopia and worst with high hyperopia, although all mean absolute differences were within 0.38 D. Similar trends were seen in the validation dataset (Table 4).

**Table 4.** Difference and Absolute Difference Between Predicted and Observed Cycloplegic Refractive Error by Age for the Best Performing Machine Learning Model XGBoost in the Training Dataset and Validation Dataset

	Training Dataset (N = 1938 Students, 3876 Eyes)				Validation Dataset (N = 1476 Students, 2951 Eyes)			
	Eyes	Observed	Difference (Predicted − Observed)	Absolute Value of Difference (Predicted − Observed)	Eyes	Observed	Difference (Predicted − Observed)	Absolute Value of Difference (Predicted − Observed)
Overall	3876	−0.07 (2.11)	0.00 (0.34)	0.26 (0.23)	2951	−0.35 (2.26)	0.07 (0.57)	0.39 (0.42)
By age (years)								
≤6	928	1.43 (0.87)	−0.01 (0.37)	0.29 (0.24)	496	1.25 (0.91)	0.16 (0.67)	0.47 (0.50)
7	634	0.96 (0.99)	0.01 (0.36)	0.28 (0.23)	526	0.95 (1.06)	0.07 (0.56)	0.39 (0.41)
8	420	0.65 (0.92)	−0.02 (0.34)	0.26 (0.22)	431	0.39 (1.24)	0.05 (0.53)	0.38 (0.37)
9	296	0.13 (1.16)	0.03 (0.34)	0.26 (0.22)	290	0.15 (1.43)	0.01 (0.55)	0.37 (0.41)
10	264	−0.21 (1.53)	0.01 (0.31)	0.24 (0.20)	138	−0.12 (1.74)	0.05 (0.59)	0.40 (0.44)
11	296	−0.34 (1.59)	−0.02 (0.32)	0.24 (0.22)	182	−1.15 (2.04)	0.05 (0.54)	0.39 (0.38)
12	218	−1.54 (2.17)	0.04 (0.37)	0.26 (0.27)	162	−1.50 (1.71)	0.09 (0.56)	0.38 (0.43)
13	164	−1.69 (2.05)	0.00 (0.27)	0.21 (0.17)	166	−1.28 (2.02)	−0.08 (0.44)	0.30 (0.32)
14	138	−1.96 (2.22)	0.00 (0.29)	0.22 (0.18)	114	−2.25 (2.09)	0.07 (0.55)	0.38 (0.40)
15	130	−2.65 (2.61)	0.02 (0.34)	0.23 (0.25)	108	−2.60 (2.00)	0.08 (0.65)	0.40 (0.52)
16	144	−3.00 (2.42)	0.07 (0.32)	0.21 (0.25)	114	−3.20 (0.68)	0.08 (0.48)	0.33 (0.36)
17	122	−2.88 (2.32)	−0.03 (0.31)	0.21 (0.23)	116	−3.80 (2.78)	0.15 (0.53)	0.38 (0.39)
18	122	−2.98 (2.32)	0.02 (0.30)	0.23 (0.20)	108	−3.10 (2.75)	0.07 (0.65)	0.43 (0.49)
By cycloplegic SER								
≤−6.0	67	−7.27 (1.54)	0.16 (0.21)	0.23 (0.27)	72	−7.59 (1.59)	0.28 (0.45)	0.40 (0.35)
>−6.0, ≤−3.0	371	−4.12 (0.81)	0.08 (0.27)	0.21 (0.19)	321	−4.14 (0.83)	0.18 (0.52)	0.37 (0.41)
>−3.0, ≤−0.5	711	−1.54 (0.72)	0.06 (0.31)	0.22 (0.23)	686	−1.54 (0.71)	0.18 (0.61)	0.38 (0.50)
>−0.5, ≤0.5	739	0.16 (0.28)	0.18 (0.34)	0.29 (0.25)	556	0.13 (0.28)	0.23 (0.48)	0.40 (0.35)
>0.5, ≤3.0	1930	1.26 (0.49)	−0.09 (0.33)	0.26 (0.21)	1271	1.29 (0.53)	−0.07 (0.51)	0.37 (0.35)
>3.0	58	4.78 (1.42)	−0.32 (0.41)	0.38 (0.36)	45	4.17 (0.87)	−0.72 (1.33)	1.15 (0.98)

Data are presented as mean (SD).

### The Best-Performing Models for Predicting Myopia Prevalence Rate by Age Group

We further evaluated the best-performing models (XGBoost, random forest) for predicting the myopia prevalence rate overall and by age group. The XGBoost model was selected because it performed best for predicting cycloplegic SER, and the predicted cycloplegic SER can be used to determine myopia status of a student. The random forest was selected because it performed best for predicting myopia status.

Using the XGBoost predicted cycloplegic SER ≤−0.5 D in either eye to define myopia, the predicted overall myopia prevalence rate was 32.1% which was similar to the observed myopia prevalence rate of 33.6% in the training dataset (Table 5). The predicted myopia prevalence rate was similar to the observed rate in each age group, with differences ≤6% (Fig.). Random forest model provided even better prediction than XGBoost with predicted myopia prevalence rate closer to the observed myopia prevalence rate overall (32.8% predicted vs. 33.6% observed), and in each age group (differences in myopia prevalence rate all ≤3%, Fig.). Similar trends were seen in the validation dataset (Table 5, Fig.).

**Table 5.** Predicted Myopia Prevalence Rate by Age Based on the Best-Performing Model for Cycloplegic SER and the Best-Performing Model for Predicting Myopia Status in Training and Validation Datasets

	Training Dataset (N = 1938 Students, 3876 Eyes)				Myopia: Validation Dataset (N = 1476 Students, 2951 Eyes)			
	n	Myopia (%): Observed	Myopia: XGBoost (Predicted by Cycloplegic SER)^*^	Myopia: Random Forest (Predicted by Myopia)	n	Myopia: Observed	Myopia: XGBoost (Predicted by Cycloplegic SER)^*^	Myopia: Random Forest (Predicted by Myopia)
Overall	1938	652 (33.6%)	623 (32.1%)	635 (32.8%)	1476	606 (41.1%)	576 (39.0%)	586 (39.7%)
By age (years)								
≤6	464	3 (0.6%)	1 (0.2%)	1 (0.2%)	248	10 (4.0%)	4 (1.6%)	7 (2.8%)
7	317	16 (5.0%)	12 (3.8%)	13 (4.1%)	263	26 (9.9%)	17 (6.5%)	15 (5.7%)
8	210	28 (13.3%)	24 (11.4%)	26 (12.4%)	216	55 (25.5%)	47 (21.8%)	51 (23.6%)
9	148	47 (31.8%)	39 (26.4%)	43 (29.1%)	145	58 (40.0%)	52 (35.9%)	56 (38.6%)
10	132	53 (40.2%)	48 (36.4%)	50 (37.9%)	69	25 (36.2%)	24 (34.8%)	24 (34.8%)
11	148	78 (52.7%)	74 (50.0%)	75 (50.7%)	91	57 (62.6%)	57 (62.6%)	57 (62.6%)
12	109	77 (70.6%)	75 (68.8%)	76 (69.7%)	81	65 (80.2%)	65 (80.2%)	65 (80.2%)
13	82	62 (75.6%)	63 (76.8%)	63 (76.8%)	83	57 (68.7%)	59 (71.1%)	58 (69.9%)
14	69	54 (78.3%)	54 (78.3%)	54 (78.3%)	57	50 (87.7%)	50 (87.7%)	50 (87.7%)
15	65	57 (87.7%)	58 (89.2%)	58 (89.2%)	54	49 (90.7%)	49 (90.7%)	50 (92.6%)
16	72	69 (95.8%)	68 (94.4%)	67 (93.1%)	57	51 (89.5%)	50 (87.7%)	51 (89.5%)
17	61	55 (90.2%)	55 (90.2%)	56 (91.8%)	58	54 (93.1%)	55 (94.8%)	55 (94.8%)
18	61	53 (86.9%)	52 (85.2%)	53 (86.9%)	54	49 (90.7%)	47 (87.0%)	47 (87.0%)

* Myopia was defined as predicted cycloplegic SER ≤−0.5 diopter in either eye.

**Figure.**
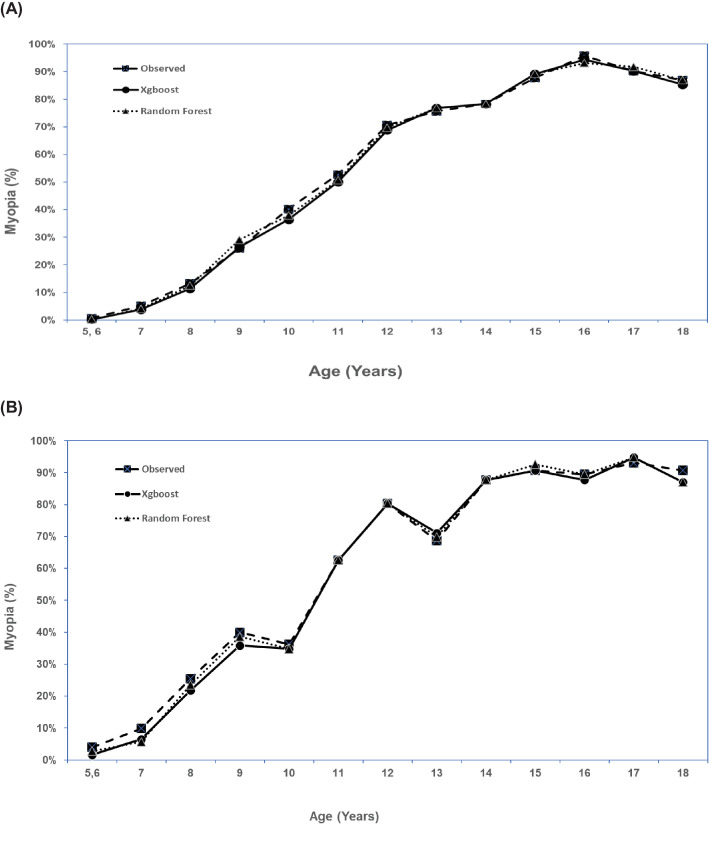
The observed and predicted prevalence rate of myopia from best performing machine learning models in each age group in the training dataset (**A**), and in the validation dataset (**B**).

### Sensitivity and Specificity of the Best-Performing Models for Predicting Myopia Status

The XGBoost had sensitivity of 94.2% (95% CI, 92.1%–95.8%) and specificity of 99.3% (95% CI, 98.8%–99.8%) in the training dataset, and similarly high in the validation dataset: sensitivity 91.1% (95% CI, 88.5%–93.2%), specificity 97.2% (95% CI, 95.9%–98.2%; Table 6). When the performance of the XGBoost was evaluated for predicting the severity of myopia, the XGBoost had a sensitivity of 95.0% (95% CI, 83.1%–99.4%) for high myopia (≤−6.0 D), 93.3% (95% CI, 91.3%–95.3%) for mild to moderate myopia (>−6.0, ≤−0.5 D), and a specificity of 99.3% (95% CI, 98.8%-99.8%) in the training dataset. In the validation dataset, the XGBoost had a sensitivity of 89.6% (95% CI, 77.3%–99.5%) for high myopia, 90.0% (87.2%–92.3%) for mild to moderate hyperopia, and a specificity of 97.2% (95% CI, 95.9%–98.2%) in the validation dataset. The random forest model yielded sensitivity of 95.7% (95% CI, 93.9%–97.1%) and specificity of 99.1% (95% CI, 98.5%–99.6%) in the training dataset and similarly high in the validation dataset: sensitivity 92.2% (95% CI, 89.9%–94.2%), specificity 96.9% (95% CI, 95.5%–98.0%; Table 6). When stratified by age group, the sensitivity and specificity remained high in both training and validation datasets (online Supplementary Table S7).

**Table 6.** Sensitivity and Specificity for Predicting Myopia^*^

	Training Dataset (N = 1938 Students, 3876 Eyes)		Validation Dataset (N = 1476 Students, 2951 Eyes)	
Models	Sensitivity (95% CI)	Specificity (95% CI)	Sensitivity (95% CI)	Specificity (95% CI)
Using predicted SER ≤−0.5 D from XGBoost	94.2% (92.1%, 95.8%)	99.3% (98.8%, 99.8%)	91.1% (88.5%, 93.2%)	97.2% (95.9%, 98.2%)
Using predicted myopia yes/no from random forest	95.7% (93.9%, 97.1%)	99.1% (98.5%, 99.6%)	92.2% (89.9%, 94.2%)	96.9% (95.5%, 98.0%)

* Myopia based on predicted cycloplegic SER (XGboost model) or myopia status yes/no (random forest model).

## Discussion

Using datasets from a large school-based myopia study, we developed and validated several ML models for predicting cycloplegic SER and myopia status based on the noncycloplegic clinical data. When the cycloplegic refractive error cannot be obtained, ML models have the potential to be used to estimate cycloplegic refractive error of an individual child as well as to determine the myopia prevalence rate in a specific population. In both the training and validation datasets, we found that ML models performed very well in predicting cycloplegic SER and myopia status. The XGBoost model performed best for predicting cycloplegic SER and the random forest model performed best or predicting myopia status. With high sensitivity (>91%) and specificity (>96%), these best-performing models accurately predicted myopia prevalence rate in all age groups.

Using the same datasets as this study, we previously developed and validated a traditional regression-based prediction model for predicting cycloplegic SER.^29^ The model had a sensitivity of 87% and specificity of 98% in the training dataset and sensitivity of 84% and specificity of 98% in the validation dataset for detecting myopia. The combination of predicted cycloplegic SER from the traditional regression-based prediction model and UCVA (i.e., defining myopia positive as SER ≤−0.5 D or UCVA 20/40 or worse in either eye) increased sensitivity and specificity, though the ML models discussed here outperformed our previous regression-based prediction models. This improved prediction performance may be due to the power of ML models to accommodate complex nonlinear relationships between predictors and refractive error outcomes. These findings support that ML models can be useful in epidemiological studies of myopia in children, particularly when administering cycloplegic agents is not feasible.

For our ML models, we considered ocular biometric measures that can be reliably measured without cycloplegia, demographics, and other easily obtainable measures such as UCVA. Based on the feature importance analysis, we found that the noncycloplegic SER, AL, AL/CR ratio and UCVA were consistently among the top four important features for predicting cycloplegic SER. These measures are well known to be associated with cycloplegic refractive error and have been used for predicting cycloplegic refractive error in prior studies.^12^^,^^19^^,^^20^^,^^28^^,^^29^

Several studies have attempted to detect myopia in children without the application of cycloplegic eyedrops using statistical prediction models.^15^^–^^22^ These statistical models explored the prediction of cycloplegic refractive error using various predictors including ocular biometric measures obtained under noncycloplegic conditions,^19^ noncycloplegic refractive error and UCVA,^21^ a combination of ocular biometric measures, noncycloplegic refractive error and UCVA.^29^ Given the variability in study design, previous studies yielded mixed results, with *R*^2^ ranging from 0.26 to 0.93. In particular, Sankaridurg et al.^21^ reported a regression-based prediction model that used age, UCVA, and noncycloplegic refractive error for predicting cycloplegic refractive error in a total of 6825 Chinese children aged four to 15 years. The model yielded *R*^2^ of 0.91, a sensitivity of 89.3% and a specificity of 97.6% for predicting myopia. The regression model developed specifically for children with UCVA worse than 6/6 yielded *R*^2^ of 0.93 with a sensitivity of 89.5% and a specificity of 97.4%.^21^ In comparison to previous studies using regression-based predictions, our study is the first to apply modern ML models that considered comprehensive predictors which are all readily obtainable without cycloplegia. Our ML models yielded better performance than previous models. Because our ML models were developed in a large sample and independently validated in another large sample of children of many ages (5 to 18 years old) with varied refractive error status (from −14.1 to 8.4 D), our model has great potential to be applicable to population-based myopia research, when measuring cycloplegic refractive error in all children is not feasible.

In this study, we developed ML models for predicting both cycloplegic refractive error (a continuous variable) and myopia status (a binary variable). Predicting cycloplegic refractive error can be useful both for detecting myopia and quantifying myopia severity, because the predicted cycloplegic SER (a continuous measure) can be later used to define the presence of myopia (e.g., predicted cycloplegic SER ≤−0.5 D as myopia) and to determine the severity of myopia based on the magnitude of the predicted cycloplegic SER. Among the 6 ML models for predicting cycloplegic SER, we found the XGBoost performed best when considering its performance in the validation dataset. This model not only performed well for predicting cycloplegic SER (*R*^2^ = 0.974, MAE = 0.256 D in training; *R*^2^ = 0.935, MAE = 0.393 D in validation), it also yielded high sensitivity (94% in training, 91% in validation) and specificity (99% in training, 97% in validation) for detecting myopia.

Estimating the prevalence rate of myopia is the primary interest of many large epidemiological studies where defining myopia using cycloplegic refractive error may not be feasible. To address this, we evaluated 4 ML models for directly predicting myopia status and found that the random forest model performed best. The random forest model provided higher sensitivity (with similar specificity) and more accurate prediction of myopia prevalence rate than using the XGBoost predicted cycloplegic SER. The myopia predicted prevalence rates from both XGBoost and random forest were very close to the observed myopia prevalence rate, supporting their potential use in the future epidemiological studies for estimating myopia rate. Because refractive error changes with age, we evaluated the performance of ML models for each age group and found that ML models performed consistently well across all groups five to 18 years, with small differences between predicted and observed myopia rate. These robust results support the use of ML models for predicting refractive error or myopia status in children of various ages.

The generalizability of our study may be slightly limited because of its design of using 0.5% tropicamide for cycloplegia, a NIDEK autorefractor, and an all-Chinese participant population. Thus the findings from this study may not be directly generalizable to other cycloplegic agents, types of autorefractors, or other races/ethnicities. These ML prediction models would likely require additional training before being employed in other settings. In this cross-sectional study, we were only able to evaluate the ML models for predicting the cycloplegic refractive error at a single time point. Future longitudinal studies are needed to evaluate how these ML models perform for monitoring the progression of refractive error over time or other cycloplegic medications.

In conclusion, we developed and validated several ML models for predicting cycloplegic refractive error and myopia status using readily available demographics and noncycloplegic measurements. These ML models (particularly the XGBoost and random forest) may help address epidemiological concerns about study attrition due to refusal of participation with cycloplegic eyedrops. The application of our ML models may provide more accurate estimates of myopia prevalence rate, severity of myopia and better determination of risk factors of myopia than noncycloplegic refractive error.

## Supplementary Material

Supplement 1
