# Supplementary material for: Machine Learning Models for Predicting Cycloplegic Refractive Error and Myopia Status Based on Non-Cycloplegic Data in Chinese Students
Source: Transl Vis Sci Technol. 2024 Aug 9;13(8):16. doi: 10.1167/tvst.13.8.16 (PMC11318358; doi:10.1167/tvst.13.8.16)

Table 7 (online supplement): Sensitivity and specificity for predicting myopia from the best-performing machine learning model (the random forest model) in the training and validation datasets.

	Training dataset (N=1938 Students, 3876 eyes)		Validation dataset (N=1476 students, 2951 eyes)	
	**Sensitivity (95% CI)**	**Specificity (95% CI)**	**Sensitivity (95% CI)**	**Specificity (95% CI)**
**By age (years)**				
5-8	83.0% (69.2%, 92.2%)	99.9% (99.7%, 100%)	73.6% (63.5%, 82.3%)	99.1% (98.0%, 99.7%)
9	89.4% (76.9%, 96.5%)	99.0% (94.6%, 100%)	87.9% (76.7%, 95.0%)	94.3% (87.1%, 98.1%)
10	92.5% (81.8%, 98.0%)	98.7% (93.2%, 100%)	92.0% (74.0%, 99.0%)	97.7% (88.0%, 99.9%)
11	93.6% (85.6%, 97.9%)	97.1% (90.1%, 99.7%)	91.2% (83.9%, 98.6%)	85.3% (68.9%, 95.1%)
12	97.4% (91.0%, 99.6%)	96.9% (83.8%, 99.9%)	98.5% (91.7%, 100%)	93.8% (70.0%, 99.8%)
13	98.4% (91.3%, 100%)	90.0% (68.3%, 98.8%)	94.7% (85.4%, 98.9%)	84.6% (65.1%, 95.6%)
14	98.2% (90.1%, 100%)	93.3% (68.1%, 99.8%)	98.0% (89.3%, 99.9%)	85.7% (42.1%, 99.6%)
15	100% (93.7%, 100%)	87.5% (64.6%, 100%)	100% (92.8%, 100%)	80.0% (28.4%, 99.5%)
16	97.1% (90.0%, 99.6%)	100% (29.2%, 100%)	96.1% (86.5%, 99.5%)	66.7% (22.3%, 95.7%)
17	100% (93.5%, 100%)	83.3% (35.9%, 99.6%)	100% (93.4%, 100%)	75.0% (19.4%, 99.4%)
18	100% (93.3%, 100%)	100% (63.1%, 100%)	95.9% (86.0%, 99.5%)	100% (47.8%, 100%)
**Overall**	95.7% (93.9%, 97.1%)	99.1% (98.5%, 99.6%)	92.2% (89.9%, 94.2%)	96.9% (95.5%, 98.0%)

Figure 1.1 (online supplement): Scatterplot for the observed and predicted cycloplegic SER in the training dataset.

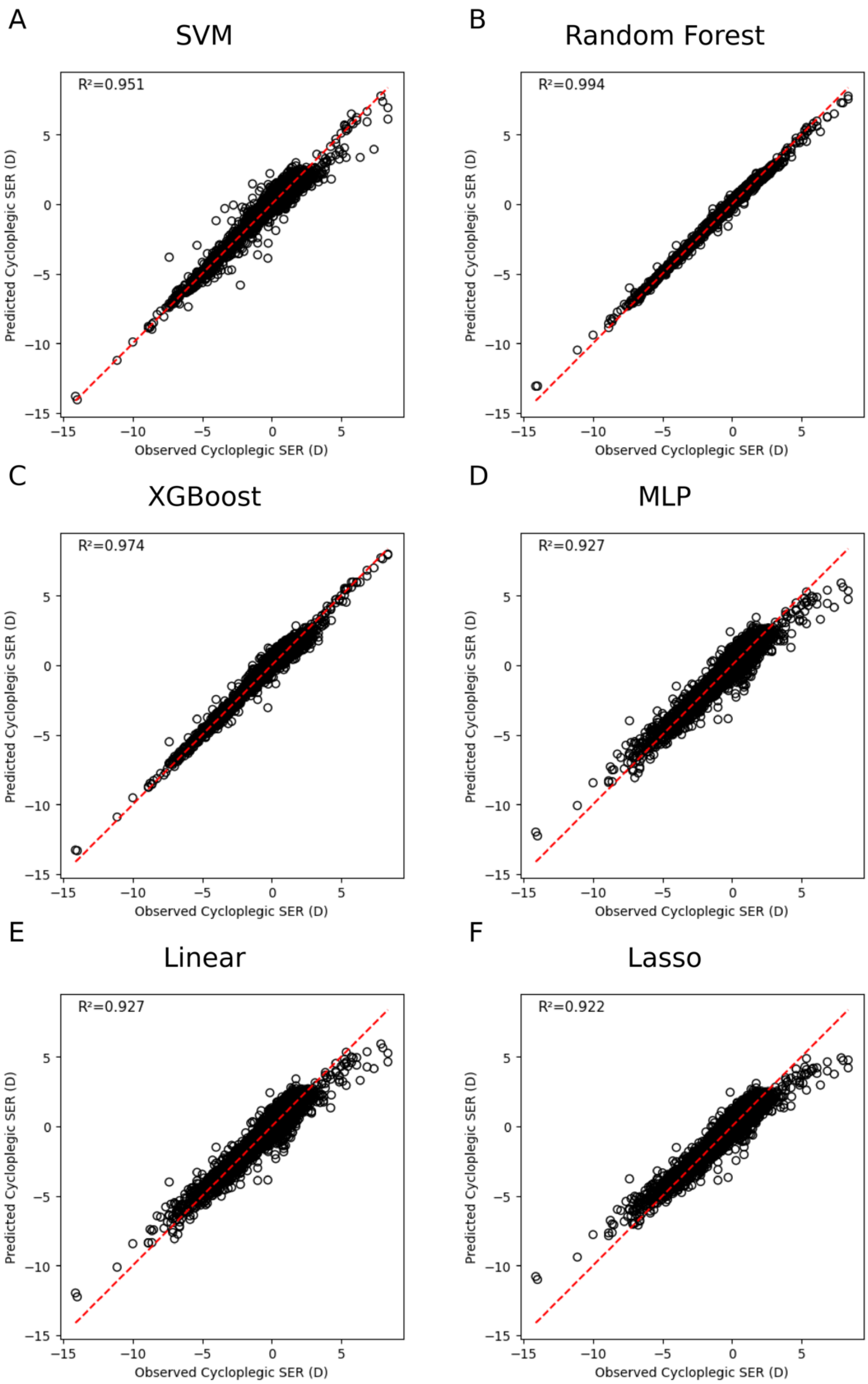

Figure 1.2 (online supplement): Scatterplot for the observed and predicted cycloplegic SER in the validation dataset.

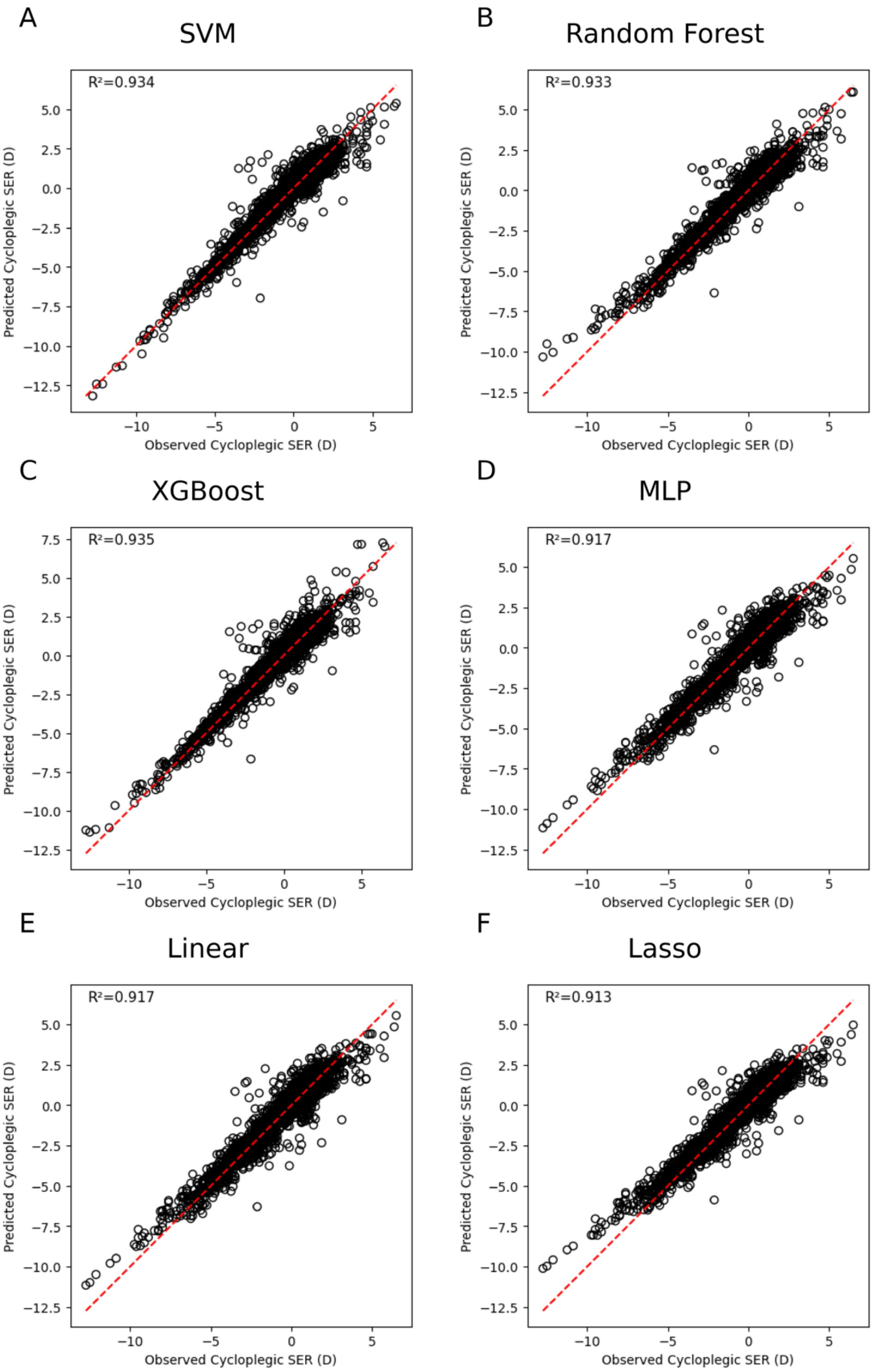
A
SVM
R²=0.934
B
Random Forest
R²=0.933
C
XGBoost
R²=0.935
D
MLP
R²=0.917
E
Linear
R²=0.917
F
Lasso
R²=0.913
Predicted Cycloplegic SER (D)
Observed Cycloplegic SER (D)

Figure 2.1 (online supplement): Feature Importance for predicting cycloplegic SER in the training dataset.

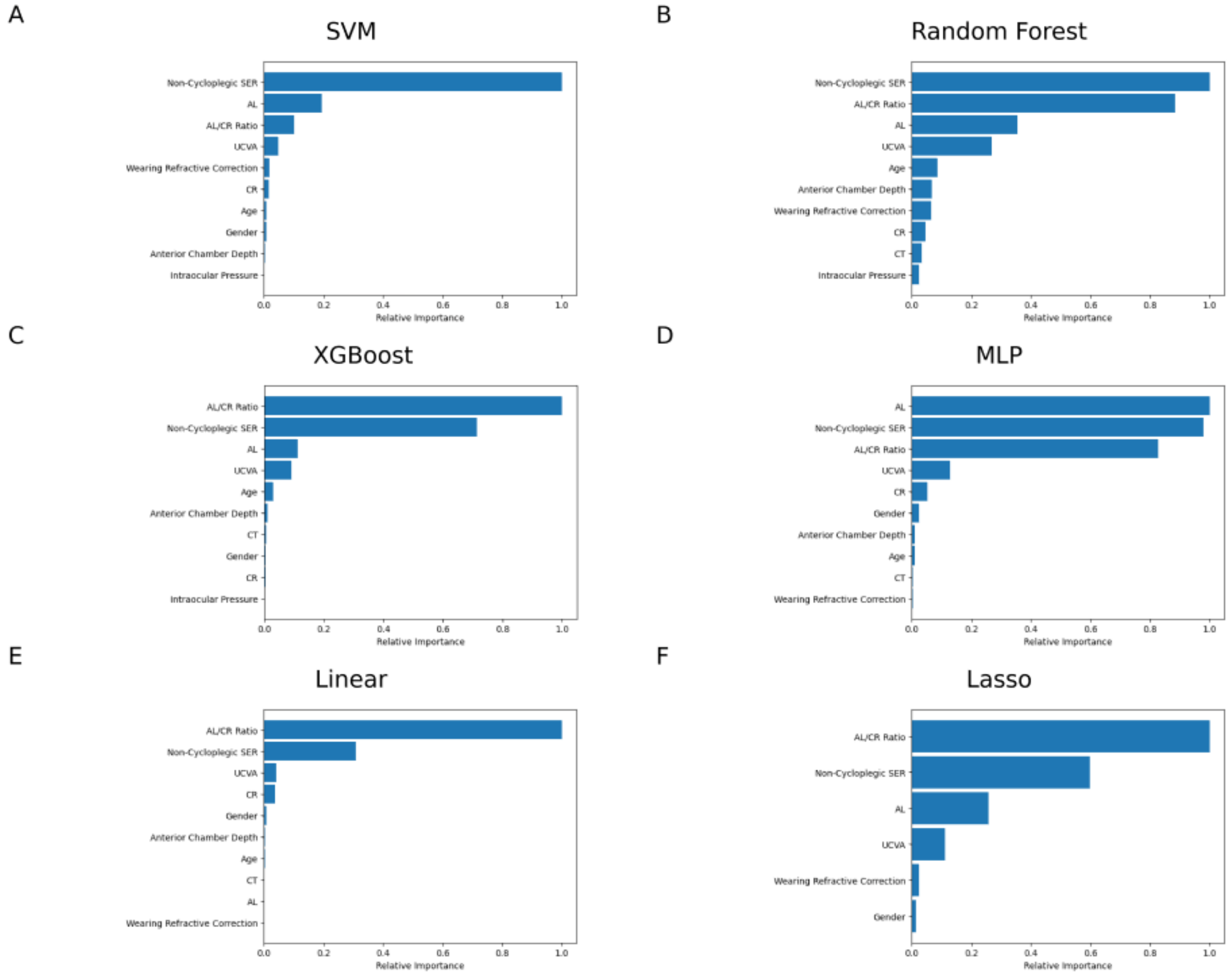

Figure 2.2 (online supplement): Feature Importance for predicting cycloplegic SER in the validation dataset.

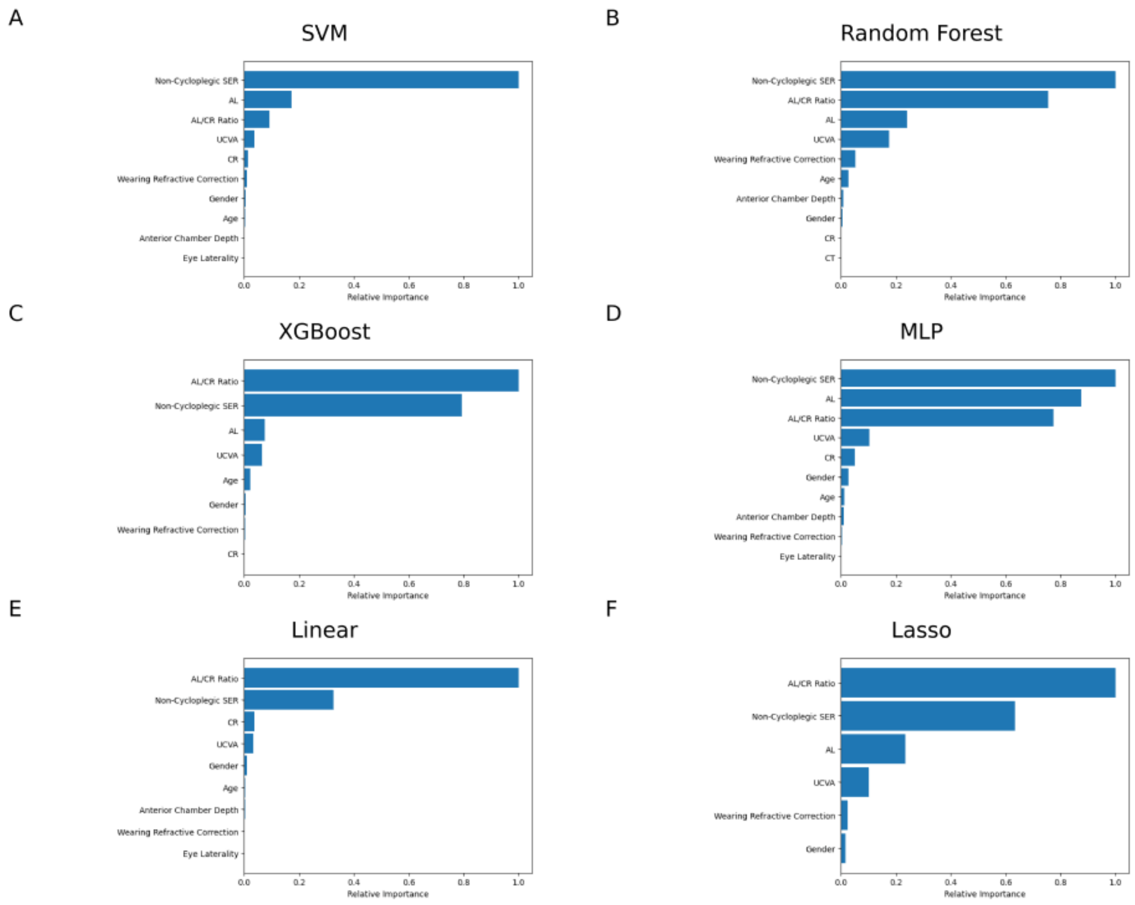

Figure 3.1 (online supplement): Receiver operating characteristic (ROC) curves for predicting myopia in the training dataset.

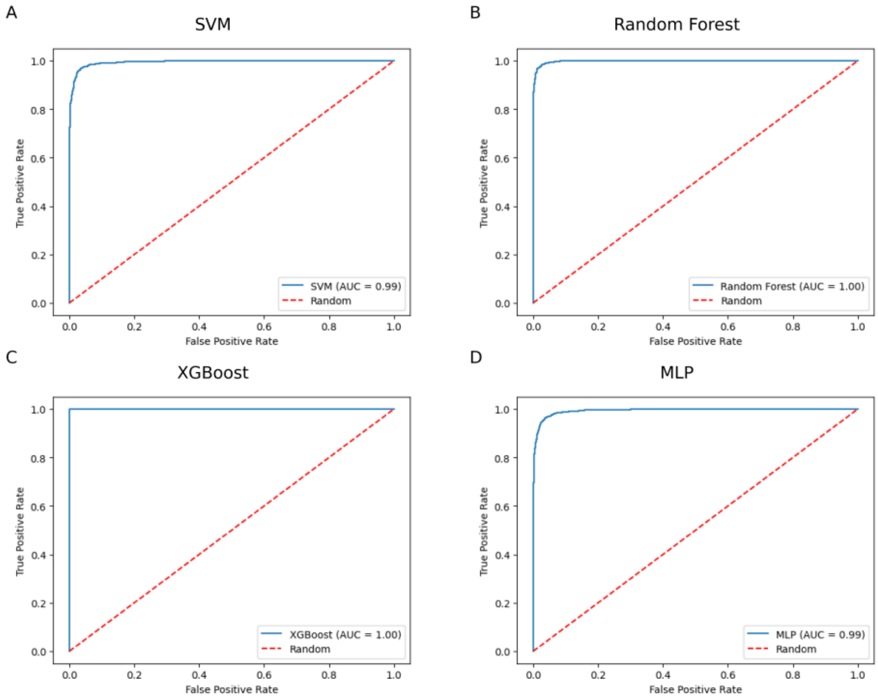

Figure 3.2 (online supplement): Receiver operating characteristic (ROC) curves for predicting myopia in the validation dataset.

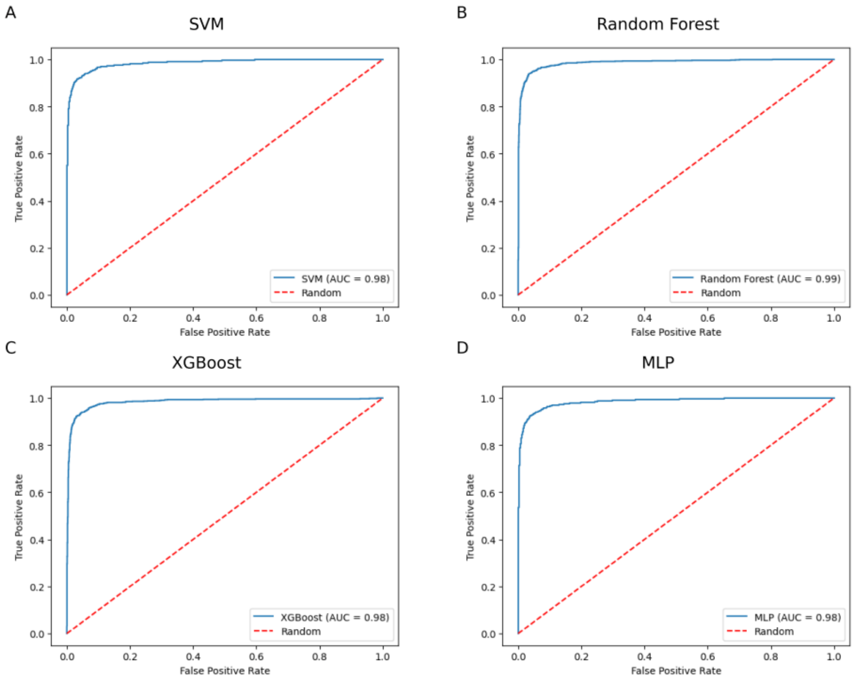

Figure 4.1 (online supplement): Feature importance for predicting myopia in the training dataset.

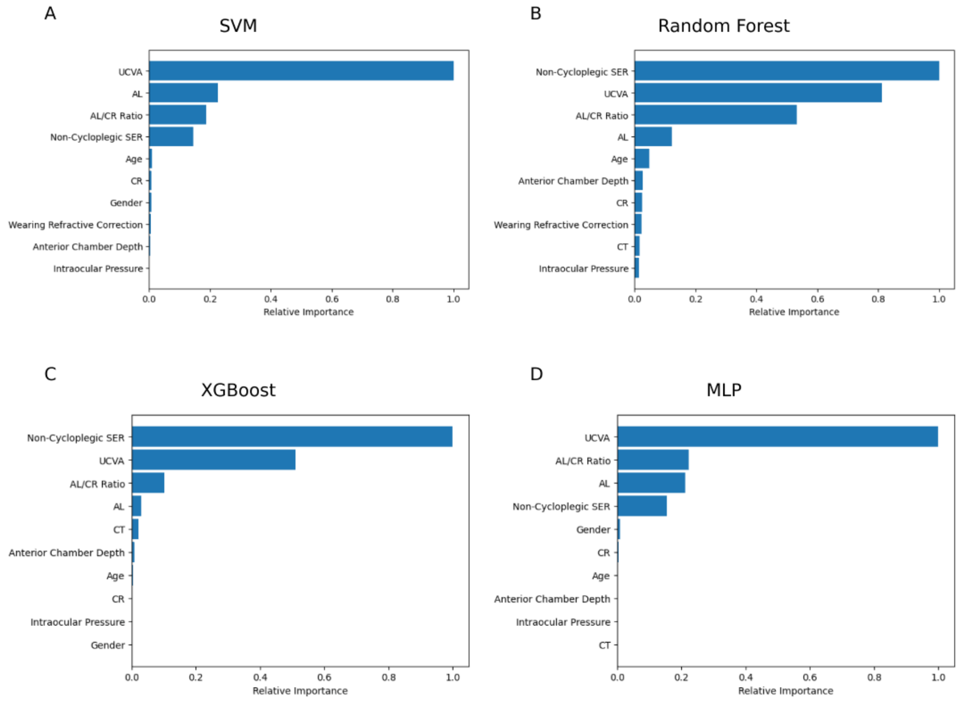

Figure 4.2 (online supplement): Feature importance for predicting myopia in the validation dataset.

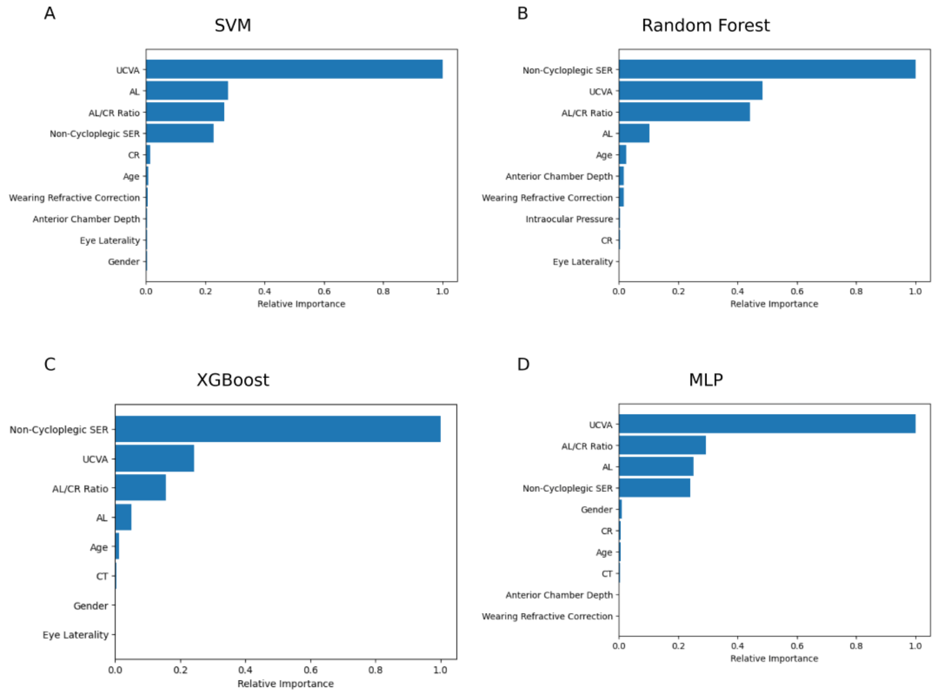

Supplement: Supplement 1 [file tvst-13-8-16_s001.pdf]
